# The Institutional Library

**Published:** 1917-03-03

**Authors:** 


### THE INSTITUTIONAL LIBRARY.

### PREVENTION AND CURE IN WARTIME.

### The Health of the Soldier.

In war various diseases which are uncommon during peace become common, and many familiar in ordinary times are seldom encountered. The aetiology, diagnosis, and treatment of disease remain unchanged, save in so far as research especially undertaken reveals new knowledge of these wartime illnesses, and save in so far as the exigencies of active service necessitate contentment with lower standards of efficiency. In Mesopotamia, for instance, in the first few weeks of the campaign in France, and in East Africa, the completeness of hospital arrangements has fallen short of the ideal; and, although such a falling short is to be avoided by all possible means and combated with all available energy, it is bound to happen in war that peace standards are unattainable.

These, then, are the main points wherein the science and art of medicine are modified during war. It may be argued that the function of preventive medicine is exalted during a campaign; to which one can only reply that, though the neglect of sanitation and hygiene is more heavily punished in war, nevertheless preventive medicine is every whit as important during peace and in civil life.

Two books recently published* bring home very clearly the special problems which confront jointly the physician and the army leader during war. The re-issue of Major Lelean's most useful book within eighteen months of the first edition shows that it has proved valuable to those for whom it was chiefly intended—namely, civilian medical officers temporarily commissioned in the Royal Army Medical Corps and unfamiliar with the special problems which confront the hygienist in war conditions. In this edition much new matter has been added; and even more than of the original issue it can be said that no medical officer attached to an infantry or any other combatant unit can do his duty by his comrades and his country unless he has absorbed and digested the details as well as the principles of military sanitation as enunciated in this book.

One could wish that the case for compulsory anti-enteric inoculation so ably put forward by Major Lelean could be illustrated by the figures and statistics from the experience of this war; such statistics will be available, doubtless, after the war, but cannot be disclosed at present for fear of aiding the enemy in his calculation of our rates of mortality. There are so many points of interest throughout this extremely practical and common-sense text-book that one hesitates to particularise amongst them. Next to the chapters on trench sanitation perhaps the most important section is that on medical organisation in the field. On one much disputed point the author hedges—namely, over the relative advantages and disadvantages of insisting on men of widely different heights using a pace of the same length when on the march. The impression is left that he agrees at heart with those who do not advocate rigid adherence to parade-ground teaching in this respect; but he is not dogmatic on the matter.

Dr. Hurst's book does not entirely exclude prophylaxis from its purview, but is chiefly concerned with disease which has already arisen rather than with methods of prevention. The opening and longest section deals with functional nervous disorders, and rightly so, for there is more misunderstanding of these troubles at home here than of any other (medical) condition for which men are invalided from the Front. There is some excuse for the public in its failure to realise that "shell shock," "nervous exhaustion," and other synonyms for the psychopathic manifestations of severe fright are largely—nay, almost wholly—functional in nature; but there is no such excuse for the medical profession, many members of which are still much too prone to intensify the troubles of such broken-down warriors by injudicious sympathy and softness. These sections are full of sound sense.

It is a pleasure also to note the firm stand which Dr. Hurst takes on the subject of tobacco-smoking in "soldier's heart." Only the other day the present writer came across a well-known knight in charge of military beds who allows his cardiac patients to smoke as many cigarettes as philanthropists and friends choose to supply. It is to be hoped this is exceptional, but it can only be described as a gross scandal. In the chapters on enteric fevers there are a few statements which will not command universal assent; possibly the enteric fevers which the author has seen at Salonika differ from those which occur in France. For example, it is not true of paratyphoid B as seen in France that the same proportion of bowel hæmorrhages is seen as in real typhoid; and it is news to the writer that there is a very marked difference in this respect between paratyphoid A and paratyphoid B. Cerebral abscess and cystitis are not mentioned in the complications of the paratyphoid fevers; both have been described since the war literature on these fevers began to appear. Splenic enlargement (palpable) has been found in a far smaller percentage of cases in France than Dr. Hurst found it in at Salonika.

Presumably "trench foot" is regarded as a surgical condition, which in severe cases it undoubtedly is; a chapter on this condition would not have been out of place in a medical text-book. Both these books are well produced, Dr. Hurst's especially, and are a credit to British medicine and to British publishers.

* *Medical Diseases of the War.* By Arthur F. Hurst, M.D., F.R.C.P., Temporary Major, R.A.M.C. London: Edwin Arnold. 1917. Pp. 151. Price 6s. net.
*Sanitation in War.* By Major P. S. Lelean, C.B., F.R.C.S., R.A.M.C. Second Edition. London: J. and A. Churchill. 1917. Pp. 336. Price 6s. net.

### THE MEDICAL INSPECTION OF SCHOOLS.*

### Its Relation to Social Efficiency.

We have perused with interest and with profit Dr. Leipoldt's article on the medical inspection of schools in relation to social efficiency, as the problem presents itself to the inspector in the Transvaal. As the writer admits, this is largely pioneer work in South Africa, and he is, of necessity, face to face with difficulties which, for the most part, have been overcome by us at home; nevertheless, there is very much in what he has to tell us which all inspectors and education authorities may well take to heart.

Starting with the assumption that where an obligation is imposed by the State, assistance must be given to those who are unable to fulfil such obligations, he accuses us of attacking the problem of medical inspection of school children in a too haphazard fashion; for, since the health of the child directly concerns the State in so far as he is a member of the community and the welfare of the State depends on the health of the children; so the responsibility of the State does not end with the inspection of children of school age; but, logically, should include children of all ages and—to go even further—every citizen. School inspection should be, in fact, a branch of a State Service of Health which controls public health measures generally.

Dr. Leipoldt lays down the requirements of a medical inspection scheme which would aim at a practical improvement of the children of a community, and he then proceeds to show us how far these principles have been carried into effect in the Transvaal.

These requirements are as follows :

(1) The co-ordination of the scheme to the existing system of education, making provision for local conditions and circumstances.

(2) Attention should be mainly concentrated upon the removal of defects discovered in children at the routine inspections.

(3) The stimulation of public and parental interest in problems of juvenile hygiene and social efficiency; and

(4) The improvement of those industrial, domestic, and economic conditions which adversely affect the rising generation.

### What Dr. Leipoldt Aims at.

Referring to these seriatim, under No. (1) the writer tells us how important he conceives it to be that medical inspection work should be centralised. He believes it to be impossible, for example, for the local authority to view such aspects of medical inspection as the investigation of and the provision for mentally defective children and the incidence of infectious disease in schools in their true perspective. In the Transvaal medical inspection of schools is an integral part of the Department of Education, the medical inspector being directly responsible for the Department. But, unfortunately, the same does not apply as yet to the Public Health Service.

Regarding No. (2), Dr. Leipoldt points out that the mere compiling of statistics dealing with defects, valuable as this may be, is of little importance compared with the steps to be taken for their removal.

Let them get to work on the removal of disease in our schools by routine inspection, by educating the parents, and by encouraging them to act. He tells us of two diseases, malaria and bilharziasis, both of which seriously affect the health of the growing children in South Africa, and in some cases undoubtedly affect the wage-earning capacity in later life. These diseases we are free from in our schools at home, but other disabilities, such as caries of the teeth, with its train of constitutional symptoms, gastro-intestinal troubles, defects of sight, all medical inspectors are well acquainted with, and these present the same problems all the world over, interfering as they do with the natural physique of our growing population. To deal with these defects in the Transvaal provision is made for the establishment of school clinics, travelling dental clinics, and a travelling hospital. School clinics cannot be utilised for the smaller areas, which can only be served by the travelling hospital. The school nurse has been found to be indispensable in any scheme for the treatment or remedying of defects.

### Civilising the Back Veld.

Turning to No. (3), the value of the creation of what is aptly described as a "health conscience" or as a "sanitary sensitiveness," especially in a new country where instruction by teaching and example is not what it is in an older civilisation, is dwelt upon. As at home, filthy children, verminous heads and bodies are to be found, together with dislike of cold water and exclusion of air and sunshine. To combat these conditions the school nurse's work and example are invaluable. It is proposed in the Transvaal to deal with this part of the work by lectures to parents and teachers, and, indirectly, by trying to influence the school children through the routine inspections; for ignorance, more than actual neglect, is responsible for much of the misery met with. But the interest of the teachers must be secured, and they must themselves be taught the elementary laws of sanitation and health before their influence can be secured.

Lastly, No. (4) requires the co-operation of the public health authorities and the investigation of public health problems. It is too soon, the writer tells us, to outline the manner in which the Transvaal can deal with the various problems in this connection. He points out that the mentally defective child at present is dealt with on purely repressive lines, as is the criminal. Special schools, farm colonies, and special legislation will all have to be considered, and the work done by and statistics collected by the school medical inspectors will help to solve the problem.

Dr. Leipoldt concludes a thoughtful and instructive paper by affirming that the percentage of military rejections for preventable and remediable defects in the Transvaal is far higher than it is in conscript armies in countries where medical inspection of schools has been in vogue for some years.

* *Medical Inspection of Schools in Relation to Social Efficiency.* By C. F. L. Leipoldt, F.R.C.S., L.R.C.P. From the *South African Journal of Science*, June 1916.

---

**Surgical Operations.** By K. K. Chatterji, F.R.C.S.I. (Calcutta: Butterworth and Co. (India), Ltd. 1916. 8vo. Pp. 238. With 26 Plates, many in Colour. Price 7s. 6d.)

This is a small and well-illustrated manual of operative surgery designed for the use of Indian students and written by one who is not quite conversant with idiomatic English. The operations are described well and carefully, but the author fails to give the detailed assistance which is required by young surgeons who are performing operations early in their career. Thus, in describing the operation for removal of the appendix, it would have been helpful to explain how the ascending colon is to be distinguished from a sagging transverse colon: in ligature of the ulnar artery it would have been wise to state the direction in which the fibres of the brachio-radialis and pronator radii teres actually run. As it stands, the book is useful for students who are engaged in a course of practical surgery. It could be made much more useful for those actually engaged in operating if the author would point out the difficulties and pitfalls attending each operation he describes.

**Surgical and Gynæcological Nursing.** By EDWARD MASON PARKER, M.D., F.A.C.S., and SCOTT DUDLEY BRECKINRIDGE, M.D., F.A.C.S. (Philadelphia and London: J. B. Lippincott Company. 8vo. Pp. 425. With 184 Illustrations. Price 10s. 6d. net.)

THERE are several good manuals of nursing, but none of them cover the ground taken by Drs. Parker and Breckinridge. The book is interesting therefore not only as showing the position of nursing in the United States at the present time, but also as pointing out the special difficulties with which the nursing profession has to contend in that country. It is written more for the nurse who has already obtained her certificate—the graduate nurse, as she is called in the States—than for those who are actually in training. A somewhat higher standard of knowledge and education is presupposed for the readers than is usual in books written for nurses in this country. The establishment of the College of Nursing in England, with a standardisation of examination for certificates, will produce in due course a class of graduate nurses in England for whom a book on these lines will be of special service. The book is divided into six parts, dealing respectively with infection; surgical pathology; the minor technique of surgical nursing such as splints, bandages, enemata, and catheters; the actual nursing of a patient; operations and the instruments used; surgical emergencies and cautions. There is an interesting chapter on Anoci-Association, which deals with Professor Crile's work on the reduction of shock as it is put to practical use at the Lakeside Hospital in Cleveland, Ohio. There are also valuable solutions of the problem of obtaining by accurate and scientific methods the fractional dose of a tabloid to be used for hypodermic injection. There is also a somewhat dangerous section on the attitude of a nurse to the surgeon, for it countenances the criticism by a nurse of the competence of the surgeon with whom or under whom she is working. The book is well written, the information is sound, and a special feature is made of the numerous illustrations, which are well rendered. Here and there are a few slips which may easily be corrected. In the list of abbreviations "ante cibum" and "post cibum" should be rendered before and after a meal; and not before and after meals; and the figure on page 120 needs re-drawing, since no reverse bandage could be applied in the manner there shown.

**Diseases of the Throat, Nose, and Ear, for Practitioners and Students** By W. G. PORTER, M.B., B.Sc., F.R.C.S. Ed. Second Edition. Revised by P. MCBRIDE, M.D. Ed., F.R.C.P. Ed., F.R.S.E. (Bristol: John Wright and Sons. 1916. Pp. xvi. and 280, with seventy-seven illustrations. Price 7s. 6d. net.)

A SECOND edition of the short manual on diseases of the throat, nose, and ear, by Mr. W. G. Porter, has appeared, having been revised during the author's absence on military service by Dr. P. McBride, who has added a summary of the newer methods of testing the vestibular apparatus and a short note on suspension-laryngoscopy.

It is impossible to include all the current knowledge on these subjects in a book of this size, for the text contains only 257 pages, but the attempt has been remarkably successful and is a model of clear and concise writing. Methods of examination or treatment which cannot be carried out by the general practitioner are not described. One cannot, however, but regret that the book had not been slightly expanded so as to include an account of laryngotomy and tracheotomy, which the practitioner may have to perform as an emergency at any time. It would also have added to the value of the section on the ear to have included a description of the after-treatment of the radical mastoid operation. Skilful after-treatment is essential to a good result; it is by no means easy, and, in the country, it must often be carried out by the family doctor. The book is well got up, the illustrations are excellent, and the index adequate. It should be of great use to the student or practitioner who requires a short and easily read work which gives a reliable grounding in these special subjects.

**Physics and Chemistry for Nurses.** By AMY ELIZABETH POPE. (London: G. P. Putnam's Sons. 1916. Med. 8vo. Pp. 444. 7s. 6d. net.)

THIS book marks a new and definite development and departure in the educational system of the nurse, and one which calls for observation and remark. The principles underlying cleaning, purifying, cooking, digestion, and metabolism generally are successively elaborated, as are also the important chemical and physical processes included in physiology, materia medica, and other studies which form part of the modern nurse's curriculum. Instead of merely stating how any process is carried out, the book explains why it is done, supplying the reason or reasons. It will be at once apparent that the knowledge thus gained, instead of being mechanical, becomes at once scientific, and therefore intelligible, and will inevitably provoke, or rather evoke, a reasoning mind in the student. Any nurse knows that sponging reduces temperature, but to be asked to explain exactly how this is brought about would puzzle not a few. The chapter on evaporation clears up the whole difficulty. There are interesting and practical chapters on the chemistry of cleaning and bleaching. The same remark holds good of the chapters on textiles, and on the spoiling, preservation, and adulteration of foods. Miss Pope has an easy style, and her methods of explaining difficult problems are so clear and practical that he who runs may easily read. The book should be in the hands of every nurse; the amount of information it contains is simply prodigious. It should be added that the illustrations are abundant.

